# Evaluation of Different Landslide Susceptibility Models for a Local Scale in the Chitral District, Northern Pakistan

**DOI:** 10.3390/s22093107

**Published:** 2022-04-19

**Authors:** Bilal Aslam, Ahsen Maqsoom, Umer Khalil, Omid Ghorbanzadeh, Thomas Blaschke, Danish Farooq, Rana Faisal Tufail, Salman Ali Suhail, Pedram Ghamisi

**Affiliations:** 1 Department of Earth Sciences, Quaid-e-Azam University, Islamabad 45320, Pakistan; bilalaslam45@gmail.com; 2 Department of Civil Engineering, COMSATS University Islamabad, Wah Cantt 47040, Pakistan; ahsen.maqsoom@ciitwah.edu.pk (A.M.); umerkhalil745@gmail.com (U.K.); danish.farooq@ciitwah.edu.pk (D.F.); faisal.tufail@ciitwah.edu.pk (R.F.T.); 3 Institute of Advanced Research in Artificial Intelligence (IARAI), Landstraßer Hauptstraße 5, 1030 Vienna, Austria; pedram.ghamisi@iarai.ac.at; 4 Department of Geoinformatics—Z_GIS, University of Salzburg, 5020 Salzburg, Austria; thomas.blaschke@sbg.ac.at; 5 Department of Civil Engineering, University of Lahore (UOL), Lahore 54590, Pakistan; salmanalisuhail@gmail.com; 6 Helmholtz-Zentrum Dresden-Rossendorf, Helmholtz Institute Freiberg for Resource Technology, 09599 Freiberg, Germany

**Keywords:** LANDSAT-8, machine learning (ML) techniques, logistic regression (LGR), linear regression (LR), support vector machines (SVM), analytical hierarchy process (AHP), landslide susceptibility maps (LSMs)

## Abstract

This work evaluates the performance of three machine learning (ML) techniques, namely logistic regression (LGR), linear regression (LR), and support vector machines (SVM), and two multi-criteria decision-making (MCDM) techniques, namely analytical hierarchy process (AHP) and the technique for order of preference by similarity to ideal solution (TOPSIS), for mapping landslide susceptibility in the Chitral district, northern Pakistan. Moreover, we create landslide inventory maps from LANDSAT-8 satellite images through the change vector analysis (CVA) change detection method. The change detection yields more than 500 landslide spots. After some manual post-processing correction, the landslide inventory spots are randomly split into two sets with a 70/30 ratio for training and validating the performance of the ML techniques. Sixteen topographical, hydrological, and geological landslide-related factors of the study area are prepared as GIS layers. They are used to produce landslide susceptibility maps (LSMs) with weighted overlay techniques using different weights of landslide-related factors. The accuracy assessment shows that the ML techniques outperform the MCDM methods, while SVM yields the highest accuracy of 88% for the resulting LSM.

## 1. Introduction

A natural hazard is a potentially destructive physical event that can cause social and economic harm, fatalities or injuries, material damage, and environmental degradation. Worldwide [1,2], landslides are considered a significant natural hazard causing extensive damage to the environment and societies [3,4]. Like other countries, Pakistan is prone to natural hazards, including landslides, due to its high precipitation, geological setting, and rugged terrain with steep slopes [5].

The foremost objective of landslide susceptibility analysis is identifying hazardous and high-risk areas, followed by appropriate actions to reduce negative impacts resulting from landslides [4,6]. The geographical information system (GIS) is widely used to document natural hazards, analyze environmental and infrastructure data, and provide hazard susceptibility maps [7,8]. The primary goals of such studies include recognizing and mapping landslide-prone zones. Landslide susceptibility assessments can be used to construct maps based on the local geo-environmental factors and the spatially explicit identification and zoning of possible landslide incidences in a particular region [9,10,11,12]. Landslide susceptibility maps (LSMs) are also important for planning and managing land use and reducing the risk of slope instability for the area under the study [12].

Various approaches for developing landslide susceptibility maps (LSMs) based on GIS have been proposed and practiced during the last few decades. Natural hazard susceptibility analysis has been carried out via different multi-criteria decision-making (MCDM) techniques [13]. The MCDM techniques run clear hierarchical decision structures that make it possible for different groups of experts in the domain and any evaluator, even non-expert participants, e.g., members of the local community, to be involved in a decision-making problem [14]. The MCDM techniques can reflect the real perception of spatial issues like any natural disaster susceptibility assessment by involving the mentioned groups via dynamic questionnaire surveys. Therefore, many landslide susceptibility studies used various MCDM techniques like the analytical hierarchical process (AHP) [15], the analytical network process [16], or the technique for order of preference by similarity to ideal solution (TOPSIS) [17]. Although the MCDM techniques have been widely used in several natural disaster susceptibility assessments, they have not always resulted in satisfactory results due to the preferences of different stakeholders [14]. 

During the last two decades, machine learning (ML) techniques have been used for natural disaster assessments like LSM [18]. Each ML technique has different advantages and shortcomings. The ML techniques are trained based on part of the inventory data set, and expert knowledge is mainly helpful for defining the optimal parameters of the ML techniques [19,20,21]. Various ML techniques have been proposed and experienced in landslide susceptibility analysis and LSM. However, the most widely used ones have been the support vector machine (SVM) [22], linear regression (LR) [23], logistic regression (LGR) [24,25,26], and multivariate regression [27,28]. Although the ML techniques are increasingly getting more popular for LSM, there is no evidence that a particular ML technique is the best for a specific natural hazard assessment or study area [29].

The ML technique of SVM is also among the methods that have been implemented for mapping landslide susceptibility. Its results have been found superior to other conventional ML methods by several studies [2,22,30]. The reason is that the workability of SVM is not affected by high dimensional data, which means that it can handle the high number of landslide conditioning factors. Numerous studies have also found the performance of LGR as quite optimal compared to other ML techniques, e.g., [31,32,33]. Moreover, numerous statistical methods have also been combined with ML approaches to develop hybrid forms, such as the rough set-SVM [34], the adaptive neuro-fuzzy inference system [35], ANN-MaxEnt-SVM [36], step-wise weight assessment ratio analysis [37], and ANN-fuzzy logic [38,39] to achieve better accuracies for the susceptibility analysis. However, more recently, deep learning algorithms such as the recurrent neural network and convolutional neural network [40,41,42,43] are also being applied for landslide susceptibility assessments. Moreover, the landslide inventory dataset plays an essential role in the training of both machine and deep learning algorithms as well as the accuracy assessment of any LSM model, such as GIS-based MCDM. Therefore, a comprehensive dataset of historical landslides is an important input for understanding the landslide susceptibility of the area under investigation [43].

The Chitral district is situated in northern Pakistan in the Hindu Kush range. Due to the rugged topography and active seismicity, the Hindu Kush range is a hazardous event hotspot. This part of northern Pakistan has recently been experiencing several landslides every year. As mentioned by Ali and Biermanns [5], aside from the general conditioning factors of lithology, relief, geological structure, and geo-mechanical properties, the conditioning factors (temperature variation, seismicity, precipitation, and active or inactive loads) are the primary cause of landslides. In most of the Chitral district (34%), the observed altitude is about 4500 m above sea level [44]. The climatic conditions of the eastern Hindu Kush are classified as high-altitude continental. There is a significant inconsistency in the annual rainfall throughout the region [45]. Glaciers and ice or snow cover more than 12% of the district permanently [46]. Roohi and Ashraf [47] documented 187 icy waters (a combination of water and ice) in the Chitral valley with an approximate area of 9 km^2^. Moreover, the increasing anthropogenic activities, the changing global climate, and the dynamic tectonic nature of the region have been causing more landslides. All these factors make our selected case study area vulnerable to landslides. Landslide susceptibility assessment and LSM provide crucial information for hazard mitigation in this area. So far, to the best of our knowledge, no study has done landslide inventory documentation and susceptibility mapping altogether in this area. In addition, no comparison studies exist for this study area and some of the datasets used in this study have never been used before in this area. Therefore, it is essential to consider the mapping accuracies of various methods for such a sensitive area and highlight the best-performing model. 

The objective of this research was to carry out a landslide susceptibility analysis of the Chitral district in northern Pakistan and compare three ML techniques: LGR, LR, and SVM, besides the two MCDM techniques: AHP and TOPSIS. The study used multiple datasets from recent sources and developed various models. Thus, this research will provide policymakers with a baseline for further studies to mitigate landslides. This research is organized as follows. In the next section, the study area including the geology and geomorphology characteristics is introduced. In Section 3, the proposed methodology for landslide inventory generation is presented in detail, together with the applied machine learning and MCDM models in the study. Results of inventory mapping, correlation analysis of the conditioning factors, and LSMs are reviewed in Section 4. In Section 5, the lesson learned is discussed.

## 2. Study Area

The Chitral district, having a latitude and longitude of 36° 15′ 0″ N and 72° 15′ 0″ E, respectively, represents most of the northern region of Pakistan’s Khyber Pakhtunkhwa province. Chitral district is based on the drainage system of the eastern Hindu Kush range. On the northeastern side of the study area, at 4500 m above sea level, lies the Karambar Glacier, from which the Chitral River commences. The Shishi River at Drosh, the Tirich-Torikho River near Buni, and the Lutkho River at Chitral are the main streams in Chitral [48].

In the northwest, the Upper Hindu Kush is separated by the Chitral tributary from the Lower Hindu Kush in the southeast. The main valley courses downwards from 2800 m above sea level in the northeast to 2000 m above sea level in the southwest over 300 km. Glaciated Mountain ranges with altitudes between 5500 m and 7700 m border the main valley on both sides. At the northwestern verge of the Chitral basin, the Upper Hind Kush reaches altitudes of 5500 m and 7500 m and is home to one of the world’s tallest summits, Tirich Mir (7706 m). At the southeastern verge of the Chitral valley, the Lower Hindu Kush reaches altitudes between 5000 m and 7000 m. In places, the local reliefs reach elevations above 4500 m, but in most areas, it is just above 2500 m [49]. 

The geology and geomorphology of the pondered area are sophisticated and are described by displacements in numerous spatial directions over time. The local geology consists of carbonate and terrigenous deposits of the Paleozoic through the Mesozoic. From the continental shelf, the Neotethys and Paleo sedimentation reveal deposits resembling fly ash of the Karakorum and Pamir Block in the north and the south with the neighboring Kohistan Magmatic Block. Two prominent stratigraphic lineaments, specifically the Tirich Mir Suture Zone and the Northern Suture Zone, detach these tectonic features. The surface marine and fluvial deposits comprise conglomerates, carbonates, and sandstones of the Cretaceous to the Tertiary. They exist in the Karakorum Block’s intraplate structural lineament, namely, the Reshun Fault Zone. Significant twists and bends embody the regional structural elements because of the Cretaceous–Tertiary orogenic movements. These consist of a ductile distortion through the conflict phase alongside the Indus Suture Zone that resulted in the advancement of intricate makeups and crustal reduction and coagulating and elevating the ground. At the same time, a brittle distortion happened alongside Northern Structure Zone, which caused feeble foliations, cleavage surfaces, longitudinal faults, and macrofolds [50].

**Figure 1:**
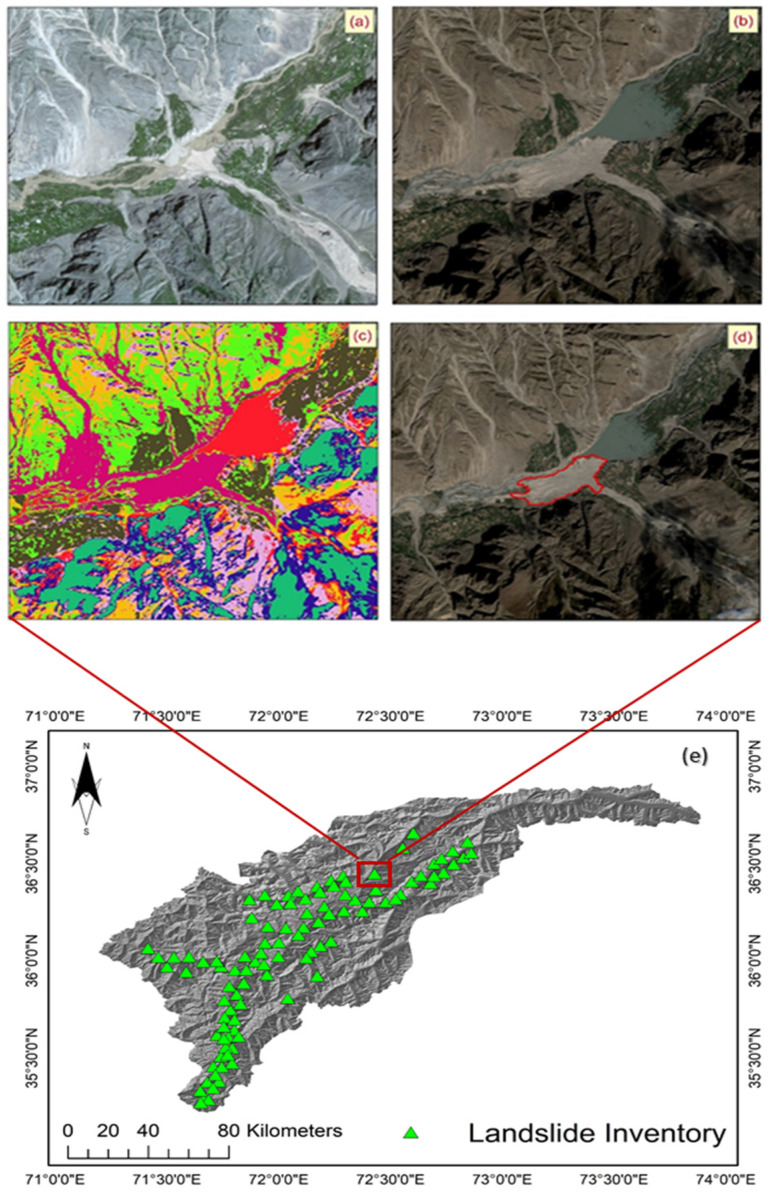

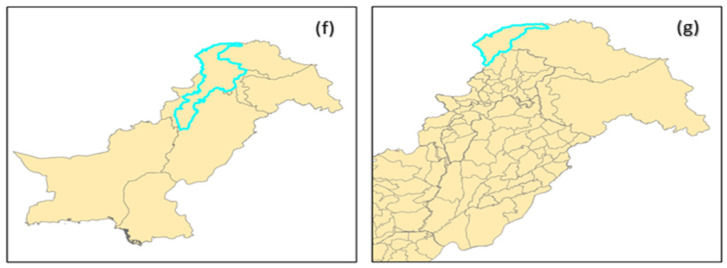
Pre-landslide imagery before a historical event, (**a**); post-landslide imagery, (**b**); image classification, (**c**); landslide identification (illustrated with red polygon), (**d**); the study area for the landslide susceptibility mapping with past landslide locations, (**e**); Pakistan with the highlighted part representing the KPK district, (**f**); districts of Pakistan with the highlighted part signifying the Chitral district, (**g**).

The regional climate is classified as semi-arid. Severe temperatures (cold and hot), low rainfall, and dampness characterize the region as arid to semi-arid. The temperature fluctuates extensively in the region and varies depending on elevation, valley winds, and orography. The mean yearly temperature is 15.6 °C, but it can drop to −1 °C in January and go up to 35 °C in July. There are dry and warm summers in the region, while with moderate to heavy rain and snow the winters are cold [49]. The region is scarcely affected by the monsoonal rainfall as it is in the rain shadow of the Himalayas [45]. The average annual rainfall in the Chitral and Drosh city areas is 450 and 600 mm. Most of this rainfall occurs in the spring and winter seasons. With a monthly rainfall of 10–25 mm, the summer and autumn are dry. At high altitudes, the combination of westerly disturbances, and in the south, the influence of marginal monsoon significantly influences the harsh infrequent summer rains [48]. Such a significant inconsistency in precipitation and temperature over different seasons of the year impacts the timely dissemination of landslide hazard in the region. The study area map is shown in Figure 1e.

## 3. Materials and Methods

**Figure 2:**
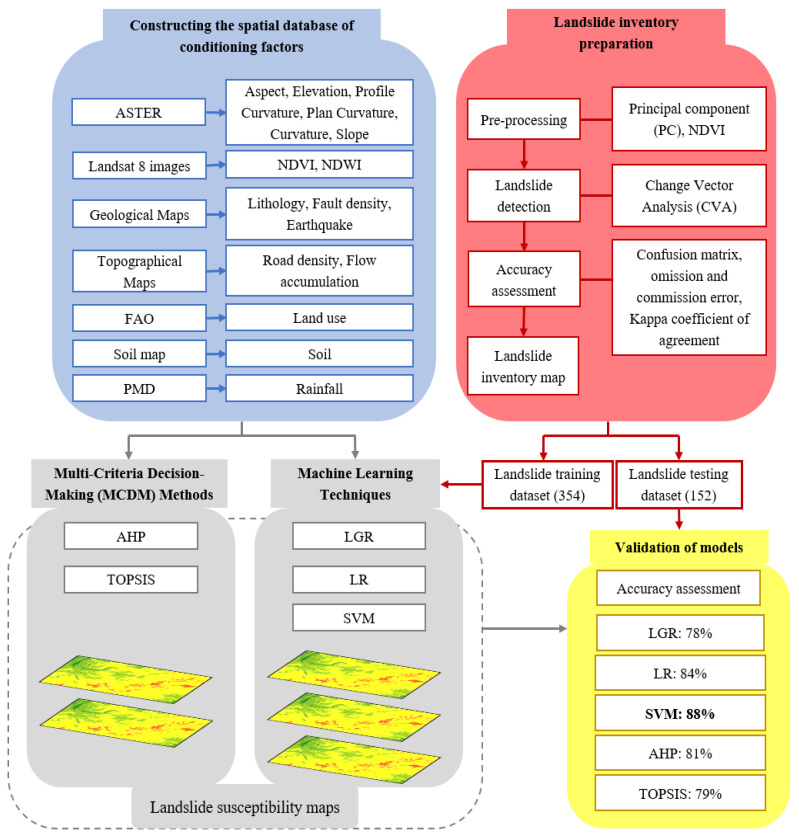
Methodology flow chart.

A landslide database for the current research was developed using satellite images, historical data, and official data from national departments. Based on the previous studies and specific study area features, sixteen conditional factors were used from topographical, hydrogeological, lithological, and geomorphological groups. Subsequently, the relative importance (weights) of the controlling elements were determined by different models. Lastly, the LSMs were developed in an ArcGIS environment by utilizing the obtained weights of the conditioning factors. A comprehensive overview of the methodology is presented in Figure 2. The step-wise procedure constituting different methods for achieving the desired task is explained.

### 3.1. Landslide Inventory

In this study, GIS was used for data collection and processing. Past landslides, including their features and locations, are generally shown in the landslide inventory maps, but the processes that activated them are not illustrated. Creating a landslide inventory map is the critical first step. The modeling approach assumes that the key to figuring out future landslide occurrences lies in the past landslides. Thus, helpful information regarding the locations of previous landslides provided by the landslide inventory map also holds the potential for identifying areas at risk of future landslides.

Consequently, the first step is to create a highly accurate landslide inventory map [41,42,43]. The landslide inventory map for this work was created using the landslide data from satellite images (LANDSAT-8) and historical records from official data from the national departments. The data highlighted 506 landslide positions (centroid) in the area. There are three different types of mass movements in the study region, namely rock fall, landslide, and debris flow. This study has processed these different types of mass movements as a single type of landslide event. The landslide inventory map of the study area is shown in Figure 1e. Automatically generating a cartographic inventory of landslides through a change detection technique is further explained.

#### 3.1.1. Pre-Processing

We used principal components (PC) analysis [51] to combine a large group of variables into a new smaller set [52] without losing the original information. As a result, the efficiency of the classification procedure is amplified by reducing the dimensionality, and the possibility of detecting differences in land cover increases. The interpretation of the variability axes of the image (LANDSAT-8 image) is facilitated by PC analysis, which allows for most of the identifying features present in the bands and others specific only to certain bands. The images taken were cloud-free and close to the landslide events. The original *m* bands were linearly combined to generate new variables (the components) in the PC analysis. Additionally, to reproduce the total variability *n*, PC is ultimately required. In a smaller number of *m* components, most of this variability is contained. As such, almost all the information is conserved; the dimensionality is reduced when the *m* bands are replaced with *n* components [53].

The used threshold methods for the change detection were statistic and secant. Initially, the threshold values were set to > 50, and the change was visualized. These values were then varied to yield the maximum change in both images [54,55]. The resulting optimal threshold values are listed in Table 1. 

The photosynthetic pigments caused a sharp absorption peak showing the vegetation in the red wavelengths. However, they exhibit strong reflection in the infrared spectrum. As the wavelength increases, the smooth monotonic increase characterizes bare soil. However, a contrary behavior is exhibited by water bodies, except for lengths corresponding to blue, where there is significant absorption at all wavelengths [56]. Based on radiometric data, it is likely to extract an index that evaluates chlorophyll density or green biomass density, as does the normalized difference vegetation index (NDVI), which is defined as a ratio of the difference between a near-infrared band and a red band, divided by their sum. 

#### 3.1.2. Change Detection

The change vector analysis (CVA) was used for change detection. The CVA model uses the magnitude and the direction of change between the images of the two dates for each co-registered pixel to define a change vector [54]. If H = (h_1_, h_2_, …, h_n_)^F^ and G = (g_1_, g_2_, …, g_n_)^F^, corresponding to two different dates (i.e., f_1_ and f_2_, the reflectance values of the pixels in two images, correspondingly, where the number of bands is given by n), then the succeeding equation can be used to determine the magnitude of the change ‖ΔG‖,

(1) $$\|\Delta G\|=\sqrt{{\left(h_{1}-g_{1}\right)}^{2}+{\left(h_{2}-g_{2}\right)}^{2}+{\left(h_{3}-g_{3}\right)}^{2}}$$

Using the characteristics of the same pixel on the two dates, the absolute magnitude of the total difference is given by ‖ΔG‖ [57]. Consequently, a high rate of change is represented by a high ‖ΔG‖, and no change is represented by a pixel with a value of ‖ΔG‖ = 0.

The geometric concept of the CVA change detection method was applied to the PC images of each of the study dates. Referring to Equation (1), the three PCs of f_2_ are represented by G, whereas the three PCs of f_1_ are represented by H. Lastly, the change map created using CVA is represented by ‖ΔG‖. Figure 1a, and b illustrates the pre- and post-landslide imagery. Figure 1c shows the change detection results, while the identified landslide is shown in Figure 1d. 

#### 3.1.3. Accuracy Assessment

From the LANDSAT-8 imagery dates, 193 polygons were sampled to gain ground–truth data based on the interpretation of colors in the satellite imagery to assess the accuracy of the change detection process. Using confusion matrices, the final thematic map produced with the unsupervised change detection method was compared with the ground–truth samples to obtain the omission–commission errors for each case.

We used the Kappa concordance coefficient of agreement [58] to compute the variance between the detected map–reality agreement in the final thematic map and the map that would be arbitrarily anticipated. Regardless of random selection, the degree of adjustment only due to the categorization accuracy is defined as an effort by the Kappa index [57]. The following Equation (2) was used to calculate the Kappa coefficient.

(2) $$K=\left(n\sum_{i=1,n}X_{ii}-\sum_{i=1,n}X_{i+}X_{+i}\right)/\left(n^{2}-\sum_{i=1,n}X_{i+}X_{+i}\right)$$

where *k*, *n*, *Xii*, and *Xi*+ *X*+*i* represent the Kappa coefficient of agreement, the sample size, the observed agreement, and the expected agreement, respectively, in each category *i*. The Kappa coefficient shows if the marked degree of agreement draws away or is not significantly different from the expected random agreement. The detected conformity highlights the diagonal of the confusion matrix, and the reality due to the randomness was calculated using the expected agreement [59].

### 3.2. The Spatial Database of Conditioning Factors

The conditioning factors in this study were chosen considering the accessible statistical data in the study area as per the study area characteristics such as topography, geological setting, etc., and the existing landslide susceptibility studies, which is a commonly applied approach. Consequently, based on former landslide susceptibility investigations [36,60,61,62,63] and examination of the characteristics of the Chitral region, the following sixteen conditioning factors, namely aspect, elevation, distance from the fault, soil, flow direction, slope, precipitation, length from the roads, the normalized difference wetness index (NDWI), land use, earthquake activity, lithology, NDVI, plane curvature, profile curvature, and curvature, were selected for mapping the landslide susceptibility of the pondered area. 

We obtained the six geomorphometric factors of the slope, aspect, elevation, and curvature from the Shuttle Radar Topography Mission (STRM) Digital Elevation Model (DEM), having a 30 m × 30 m resolution plane and profile curvature. The NDVI and NDWI were extracted using LANDSAT-8 satellite images. The infrared and red bands were used to calculate the NDVI with the formula $NDVI=\frac{IR-R}{IR+R}\;$ [64,65,66]. However, short-wave infrared and green bands were used to compute the NDWI using the formula *NDWI* = *G* − *SWIRG* + *SWIR*. The land use map was prepared using data extracted from the Food and Agricultural Organization (FAO) 2009 dataset. 

Furthermore, to create the thematic maps of earthquake activity, faults, and lithology, we used the geological maps of Pakistan at scales of 1:2,000,000. The soil map of Pakistan was used to generate the soil map of the study area. The Pakistan meteorological department (PMD) station data constructed the precipitation map. We used topographic maps and Google Earth Images to create the thematic road density map. Lastly, the data was normalized and standardized before further processing. All the maps of the landslide conditioning factors were normalized to avoid issues because of the data’s scale variability as it was acquired from different sources. All the thematic layers with different resolutions were transformed into a raster format with a resolution of 30 m × 30 m, similar to that of the derived DEM, as most of the factors were derived from DEM.

Furthermore, all the thematic layers were standardized by classifying every layer into five classes using natural break classification, and these classes were ranked from 1 (very low) to 5 (very high) based on the varying influence of each class on landslides. Ultimately, the data after normalization and standardization was checked for errors by comparing the layers with each other and looking for a difference value. The resulting difference was 0, showing no errors among the data.

### 3.3. Evaluation of Condition Factors

It is crucial to use only high-quality data when dealing with data mining methods as it directly affects prediction accuracy. The prediction process can be complex due to the high dimensionality of the training and validation sets, and the curse of dimensionality is that it always has poor prediction accuracies. Therefore, in this study, multicollinearity analysis was carried out for the selected conditioning factors to evaluate the factors so that the extraneous and redundant features could be removed.

#### Multicollinearity Analysis

Multicollinearity is a statistical trend that signifies an excellent correlation between two or more variables in a multiple regression model [62]. The present study used variance inflation factor (*VIF*) and tolerance (TOL) to spot multicollinearity among the considered conditioning factors. Suppose that a given independent variable set is defined by x = [1 × n], and *R_j_*^2^ signifies the determination coefficient when the *j*th independent variable x_j_ is regressed against all other variables in the model. The following equation gives the *VIF* value:

(3) $$VIF=\frac{1}{\left(1-R_{j}^{2}\right)}$$

The inverse of the *VIF* value gives the TOL value, and it denotes the extent of linear correlation among individual variables. As the values of TOL and *VIF* are reciprocal to each other, this means that if the value of one of these indicators is lower, subsequently, the value of the other will be higher. A *VIF* value of more than 10 or a TOL value less than 0.1 indicates that the corresponding factors have multicollinearity and should not be considered for further analysis. 

### 3.4. Training and Validation Database

Bui and Ho [58] stated that landslide and non-landslide samples are required when using data mining techniques to produce LSMs. However, the extent of landslide statistics is far less than that of non-landslide statistics, even if all landslide statistics are exploited [67]. Subsequently, in this study, along with the 506 landslide positions used to create the landslide inventory, 506 non-landslide positions were randomly selected in the area. Afterward, the landslide and the non-landslide points were randomly split with a ratio of 70/30. There are no established criteria for separating the data for susceptibility modeling. However, the ratio of 70/30 is the most practiced ratio for splitting data in the research community. Of all of the 506 landslide locations, 354 (70%) randomly sampled were used as training data to construct the models. The remaining 152 (30%) were used as testing samples for model validation. Similarly, for the non-landslide locations, 354 (70%) randomly sampled points were used for training and the remaining 152 (30%) for the model validation.

Subsequently, all landslide and non-landslide locations’ raster values in combination with the data of the 16 conditioning factors were used to build the training and validation datasets. A value of 1 was assigned to all landslide points and 0 to all non-landslide points to assemble the training and validation datasets. 

### 3.5. Machine Learning (ML) Techniques

This study used three ML techniques for mapping the landslide susceptibility of the pondered area. R-Studio was used to implement LGR, LR, and SVM to calculate the importance of each contributing factor. In a 10-fold cross-validation process, the considered conditioning factors were used to construct the landslide models. All three models had the same structure, as they are all regression models [68,69,70]. Thus, the architecture of the models included 16 input layers and 15 hidden layers. The epoch value was 150, and the loss function was the root mean square error function. The learning rate was 0.0067. The correlation value was calculated using all three models by comparing the predicted and observed outcomes in testing datasets. A higher value of correlation represents a more precise model calculation. A brief description of all ML methods used for susceptibility analysis follows. 

#### 3.5.1. Linear Regression (LR)

LR was used to define the parameters responsible for conditioning landslides in the study area and to investigate multivariate statistics. All the input datasets used in this study are used as input parameters for LR analysis, because all the considered factors contribute to landslides. The multiple linear regression method reveals how the changing standard deviation of predicators and independent variables changes landslide susceptibility. Moreover, in the study area for landslide susceptibility, this method helped make a linear function (model) and Equation (4). The theoretical model for this study is described as follows.

L = B_0_ + b_1_X_1_ + b_2_X_2_ + b_3_X_3_ + … + b_m_X_m_ + ℇ (4)

where, in each sampling unit, the occurrence of landslides is represented by L; for each mapping unit, the observed independent variables are represented by all the X’s; the B’s are the estimated coefficients (weights); and model error is represented by ℇ [71].

#### 3.5.2. Logistic Regression (LGR)

In the earth sciences, the most common statistical method used is the multiple regression method, which is articulated as the following linear Equation (5):

Y = b_0_ + b_1_x_1_ + b_2_x_2_ + … + b_n_x_n_ (5)

where the presence (1) or absence (0) of the landslide is represented by a dependent variable Y, the model’s intercept is b_0_, and b_1_ represents the partial regression coefficients… b_n_, x_1_ … x_n_, which are the independent variables.

LGR is a multivariate analysis model that is convenient for forecasting the existence or nonexistence of a representative or consequence centered on assessments of a set of predictor variables, as Lee and Sambath [72] stated. Yesilnacar and Topal [73] noted that the basis for LGR is that the dependent variable is dichotomous. In this model, the predictors of the dependent variable are the independent variables. They can be calculated on an ordinal, small, break, or fraction scale. 

#### 3.5.3. Support Vector Machine (SVM)

SVM is an ML method based on the refinement of groups in a high-dimensional attribute space created via nonlinear alterations of the predictors [74]. All considered conditional factors are used as input parameters for SVM analysis because of all the factors contributing to landslides. The basic theory of SVM is the statistical learning theory [75]. For classifying datasets, an evaluation hyperplane is computed in this high-dimensional space. For susceptibility modeling, the potentials of SVM were demonstrated by Brenning [76]. The effectiveness of working with high-dimensional and linearly non-separable datasets is the reason for the wide use of SVM in diverse applications and to deal with regression complications [77,78]. The SVM reduces both model complexity and error.

### 3.6. Multi-Criteria Decision-Making (MCDM) Techniques

#### 3.6.1. Analytical Hierarchy Process (AHP)

Saaty [79] developed the AHP method, a useful tool that uses the hierarchy principle to organize and analyze complex decisions. For the application of this method, a necessary first step is that the composite formless dilemma is broken down into its constituent aspects. These aspects are then arranged in hierarchical order. Afterward, based on the comparative status of each feature, values are allocated to subjective judgments; lastly, the decisions are integrated to decide the weightings to be allotted to these features [80]. Each factor is valued compared to every other factor while structuring a pair-wise comparison matrix by allocating an overall comparative value between 1 and 9 to the traversing cell.

During the pair-wise comparisons of the factors in the AHP method, some consistencies may typically arise, as the judgments are driven by the subjective opinions of experts from various fields. However, the AHP method integrates an operative practice for checking the logical consistency in pair-wise comparisons. The consistency ratio (CR), as suggested by Saaty [79], is a tool that determines the consistency of the matrix by using all the selected parameters and their paired comparisons [12]. The matrix results are considered satisfactory if the value of CR is less than 0.1 [77]; otherwise, the decisions need to be revised.

#### 3.6.2. The Technique for Order Preference by Similarity to Ideal Solution (TOPSIS)

Ching and Yoon [81] presented the TOPSIS method based on the Euclidean remoteness among choices and creating substitutes [82]. This method was developed to solve decision-making problems because of incompatible and non-commensurable conditions. It consists of grading the substitutes based on the degree of adequacy ranked using the distance principle. It is readily used to solve various decision-making challenges. In this technique, the extreme detachment from the negative principle result and the briefest detachment from the principle outcome are the two conditions on which the ranking of alternatives is based [83].

### 3.7. Landslide Susceptibility Mapping

According to the weights of the conditioning factors determined using various models, the raster layers were reclassified and weighted. A direct and straightforward tool to create susceptibility maps in GIS is the Weighted Overlay Method (WOM). Several researchers have used WOM to produce susceptibility maps [84,85,86,87,88]. An overlay of raster layers was utilized to create susceptibility maps with all governing aspects. The landslide susceptibility indices for all pixels in the study region were also calculated to get the area percentages under different susceptibility classes.

## 4. Results

### 4.1. Change Detection Map

The change map created using the CVA change detection technique is shown in Figure 1c. Pixels in which a change occurred between the two study dates (pre- and post-landslide dates) are characterized by the highest (in dark pink) and lowest (in red) values. The consistency of the map seems to be increased, as the map shows spatial coherency by displaying changes in identical areas. Therefore, areas that changed the exact location between two dates in satellite images were marked as landslides. The threshold values and pixel change ratio achieved for the applied technique are given in Table 1. For landslide detection, the pixel change ratio after using the method in question, along with the accuracy assessment (omission/commission error and the Kappa coefficient of agreement) of the practiced method, is presented in Table 2. 

### 4.2. Correlation Analysis of the Conditioning Factors

The predictive ability of all the considered conditioning factors was appraised by exploiting the training set based on multicollinearity analysis. Therefore, the outcomes of the multicollinearity analysis of landslide conditioning factors are listed in Table 3. None of the considered landslide conditioning factors were found to have a VIF value greater than 10 or a TOL value less than 0.1. Consequently, all of the considered factors were considered for the prediction processes. 

### 4.3. Thematic Maps of Conditioning Factors

In this research, thematic maps of sixteen landslide conditioning factors were created to determine landslide susceptibility. The factors considered were land use, slope, soil, lithology, NDWI, NDVI, rainfall, elevation, fault density, road density, earthquake activity, flow accumulation, profile curvature, plane curvature, and aspect.

#### 4.3.1. Land Use

**Figure 3:**
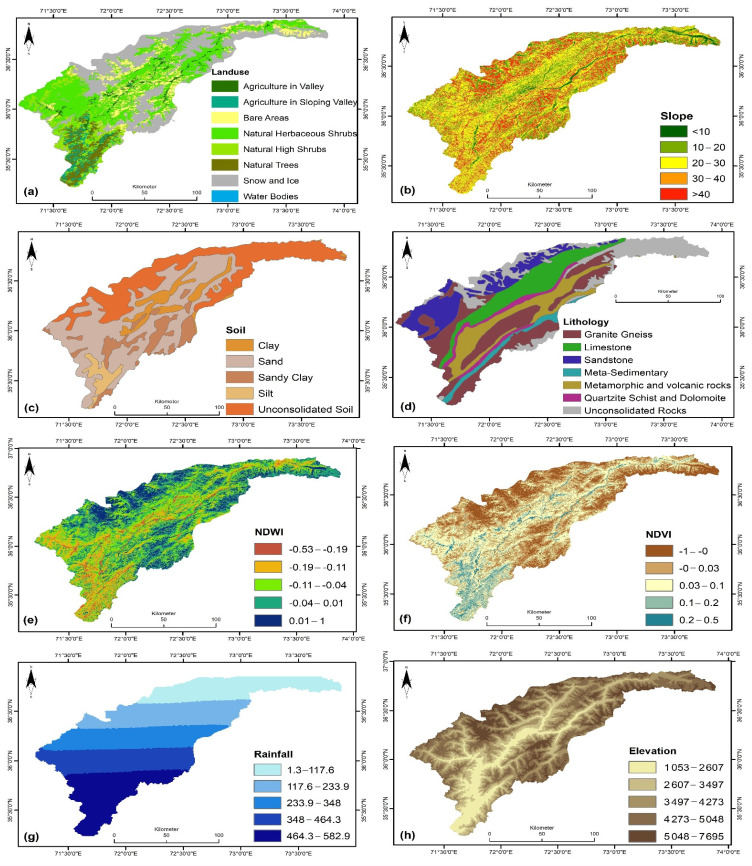

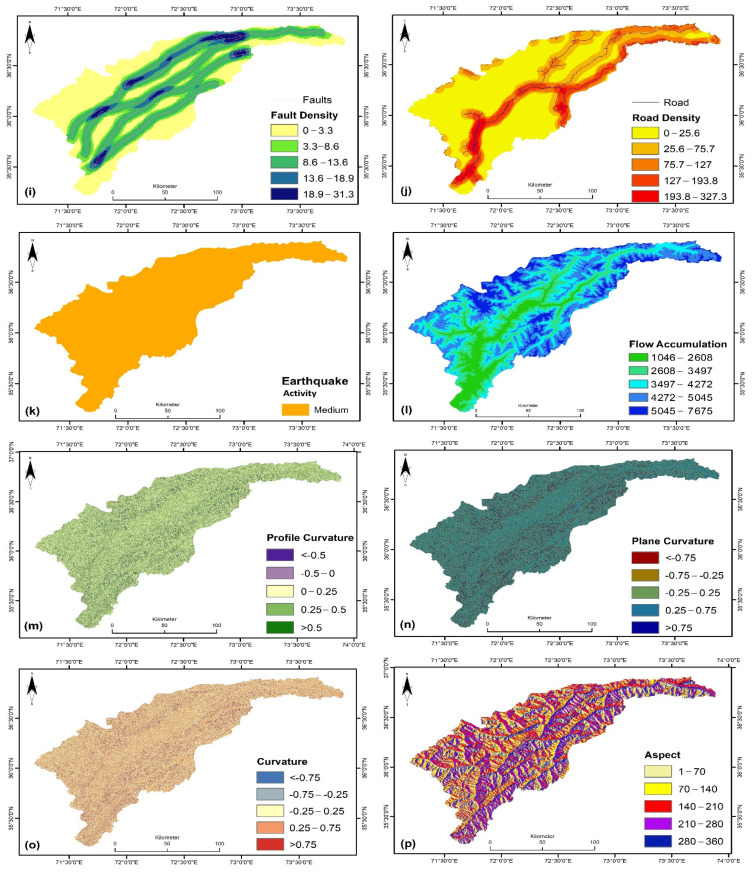
Maps of landslide conditioning factors: land use (**a**), slope (**b**), soil (**c**), lithology (**d**), NDWI (**e**), NDVI (**f**), rainfall (**g**), elevation (**h**), fault density (**i**), road density (**j**), earthquake activity (**k**), flow accumulation (**l**), profile curvature (**m**), plane curvature (**n**), curvature (**o**), and aspect (**p**).

The land use map of the study region was categorized into eight groups: agriculture in the sloping valley, agriculture in the valley, bare areas, natural herbaceous shrubs, high natural shrubs, natural trees, snow, and ice and water bodies, as illustrated in Figure 3a. Most of the considered region is covered by snow and ice and agriculture in sloping valleys. The peripheral northern and southern parts are mostly covered with snow and ice, while agriculture in sloping valleys is scattered throughout. The study area also shows a prominence of the bare regions.

#### 4.3.2. Slope

The slope of the study area was divided into five classes between < 10 and > 40. Our study area’s northern and southern peripheral parts were observed to have the steepest slopes (Figure 3b). The potential for hazards associated with slopes with steeper angles is very high, whereas it varies for the other categories.

#### 4.3.3. Soil

The soil of the Chitral district was classified into five groups, namely, clay, sand, sandy clay, silt, and unconsolidated soil (Figure 3c). The northeast region of the area is entirely composed of unconsolidated soil. The same soil type stretches to most of the region’s bordering northern and southern parts. The second most dominant soil type is sand. Clay and silt are the soil fractions that are more likely to contribute to landslides because they can absorb water, increasing the soil layer’s weight, thus increasing the chances of slope failure. 

#### 4.3.4. Lithology

A more significant part of the considered region has granite gneiss and limestone/shale, as shown in Figure 3d. The granite gneiss is a comparatively more substantial lithology than the limestone/shale, and it is less prone to landslides. The northeast region is dominated by unconsolidated lithology. In contrast, the marginal northern part is primarily composed of limestone/dolomite/sandstone, which have comparatively weaker lithologies and are highly prone to landslides. The unconsolidated lithology is composed of loosely arranged particles and thus is prone to landslide hazards.

#### 4.3.5. The Normalized Difference Wetness Index (NDWI)

The NDWI map of the area is classified between the observed minimum and maximum NDWI values, −0.53 to −1. In the study area, the regions covered with snow, either in the form of glaciers or ice, have high NDWI values. The southwest region of the study area has comparatively low NDWI values, as can be seen in Figure 3e.

#### 4.3.6. Normalized Difference Vegetation Index (NDVI)

Positive NDVI values up to 0.5, which is the maximum NDVI value in Figure 3f, correspond with healthy vegetation of varying intensities, and negative NDVI values depict no vegetation cover. Slopes with rich vegetation are less prone to landslides than those with less or no vegetation cover as they are exposed to the atmosphere and soil erosion. Most of the study area has low to moderate vegetation cover.

#### 4.3.7. Rainfall

The rainfall map presented in Figure 3g displays the scattering of rainfall between the observed maximum and minimum rainfall over the area, 1.3 mm to 582.9 mm annually. The rainfall in the area decreases gradually from south to north, as shown on the map. The high-elevation regions receive less rainfall as they generally receive snowfall. Generally, high rainfall is more likely to trigger landslides than low rainfall. 

#### 4.3.8. Elevation

The elevation map was created by classifying the observed elevation into five classes. The area’s observed high and low elevations are 1053 m to 7695 m, respectively. The higher elevation regions are in the study area’s northern, northeastern, and intermediate southern marginal regions. In contrast, the southern-most region has a low elevation, as can be seen from Figure 3h. 

#### 4.3.9. Fault Density

The region has several faults running throughout it, as presented in Figure 3i. The fault map is categorized into high and low fault density values of 0 and 31.3, respectively. The areas that are closer to faults are more prone to landslides. The high fault density regions are in the upper northeastern part and all along the central part of the study area.

#### 4.3.10. Road Density

The proximity of the areas to roads is shown as high-density values of between 127 to 327.3 in Figure 3j, whereas the rest of the area is represented by low- to medium-density values of 0 to 127. A network of roads runs through the center and mainly along the marginal south regions of the area, making these regions the ones with the highest road densities. The slopes near roads have increased proneness to landslides, and the level of hazard for the slopes away from roads gradually decreases.

#### 4.3.11. Earthquake Activity

The entire study area is characterized by medium seismicity, as depicted in the produced map shown in Figure 3k. An earthquake can influence the stability of the slope in such a way that it can result in the formation of cracks in the rocks, through which rainwater can intrude and eventually result in rock failure.

#### 4.3.12. Flow Accumulation

The study area’s flow accumulation map depicted in Figure 3l classed the area into five classes. The high and low flow accumulation density values were 1046 to 7675, respectively. The marginal northern, northwestern, and southern regions subjected to snowfall have high flow accumulation, which means they are prone to landslides. 

#### 4.3.13. Profile Curvature

The peripheral northern and southern regions have relatively moderate profile curvature in the study area, with values representing a gentle slope in the vertical plane (Figure 3m). The surfaces’ convex, concave, and horizontal characteristics are defined as the profile curvature, thus illustrating its geomorphological significance because it affects the water flow.

#### 4.3.14. Plane Curvature

As represented by the produced map illustrated in Figure 3n, the entire study area is restricted by an intermediate value of plane curvature between the two limiting values, indicating straight slopes in the horizontal plane. A positive plane curvature shows the concave surfaces, and negative plane curvature values reflect the convex surfaces. 

#### 4.3.15. Curvature

Positive curvature values represent a convex slope, negative curvature values represent a concave slope, and intermediate values represent a flat land. The research area is dominated by flat land and slightly convex slopes, as shown in Figure 3o. The marginal northern, northwestern, and southern regions have slightly convex slopes near the positive limit. 

#### 4.3.16. Aspect

The aspect map shown in Figure 3p demonstrates that most of the study area has moderate to high aspect values ranging from 140 to 280. The aspect map indicates the orientation of the slopes in the area, and the areas with greater aspect values have a comparatively high effect on the landslide hazard level and vice versa.

### 4.4. Conditioning Factor Analysis

A similar controlling element can have a different influence in various ways on landslide incidence when modeled using distinct models, as can be seen from Table 4. According to the AHP technique, slope, with a weight of 11%, is the most influencing factor for a landslide event. Other highly weighted factors include precipitation and NDWI, with 10% and 9%, respectively. According to the TOPSIS method, precipitation is the most significant factor for landslides, with a weight of 13%. Furthermore, flow and land use, both weighted at 9%, are essential factors per TOPSIS. 

The weights are derived from the landslide inventory with training datasets for the ML techniques. As per the LGR model, the NDWI, precipitation, and slope have the highest weight percentage (9% each) compared to the remaining factors. According to the LR model, the most significant factors are flow (10%), NDWI (10%), and elevation (9%). In the SVM model, flow accumulation, elevation, precipitation, and slope with their resulting weights of 9%, 9%, 11%, and 10%, respectively, are of the highest importance. Besides the factors mentioned here for each model, the rest are low or moderate-tohigh influencing factors.

### 4.5. Landslide Susceptibility Maps (LSMs)

**Figure 4:**
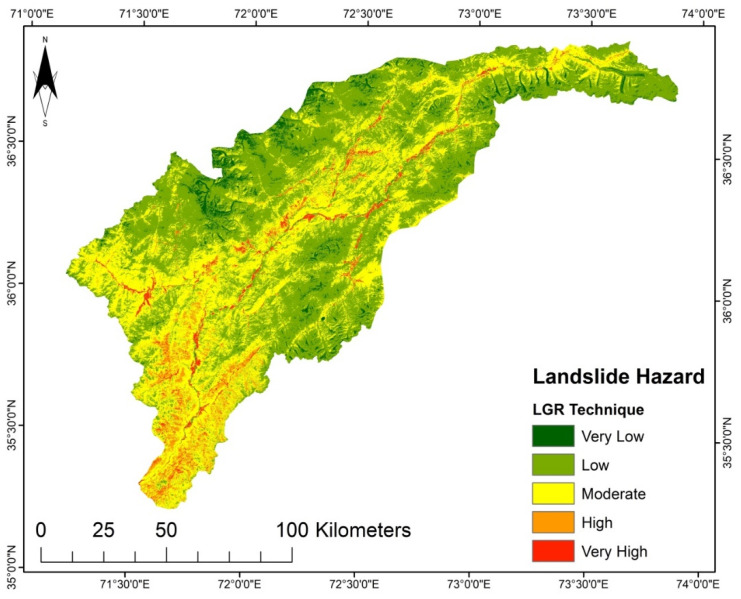
LSM from the LGR model.

**Figure 5:**
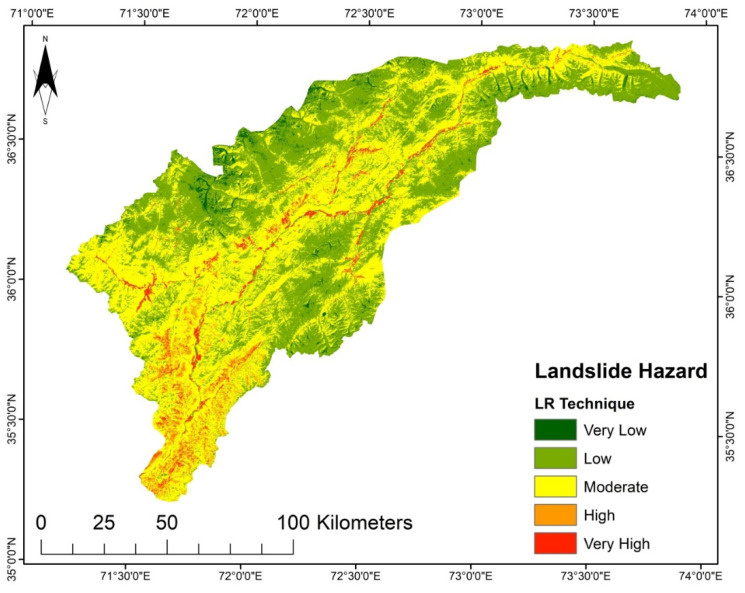
LSM from the LR model.

LSMs for the different techniques were prepared in an ArcGIS environment. They were sorted into five susceptibility groups, i.e., very high, high, moderate, low, and very low. The generated LSM presented in Figure 4, using the LGR model, reveals that the marginal areas in the north, south, and east have very low to low susceptibility. In contrast, the central and southeastern regions have areas subject to very high or high susceptibility. However, in the LSM generated using the LR model shown in Figure 5, very low and low susceptibility is found in the study area’s north, south, and northeast regions. Very high susceptibility regions are in the middle part of the district and the northwest region, which shows moderate to high susceptibility.

**Figure 6:**
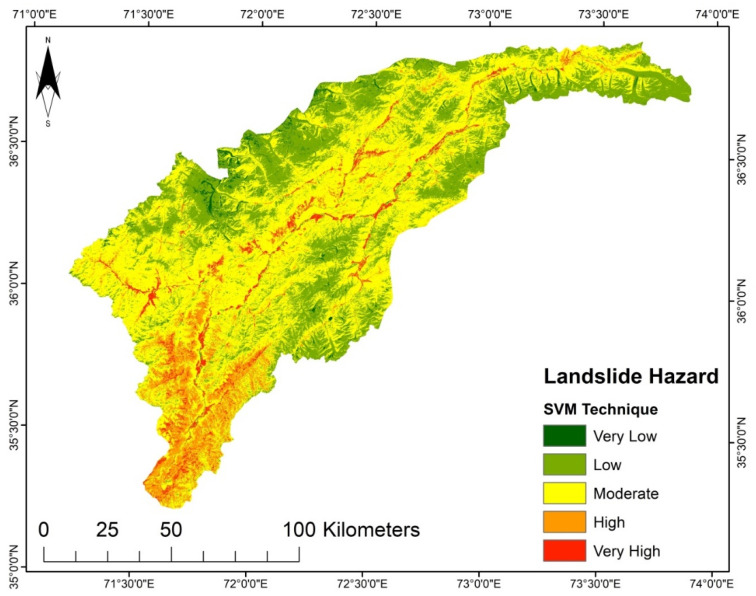
LSM from the SVM model.

Furthermore, the LSM of the study area shown in Figure 6, composed based on the SVM model, demonstrates that the southwestern region prominently exhibits high susceptibility. In contrast, most peripheral regions in the north and south show low susceptibility. Very highly susceptible regions are located throughout the central part. 

**Figure 7:**
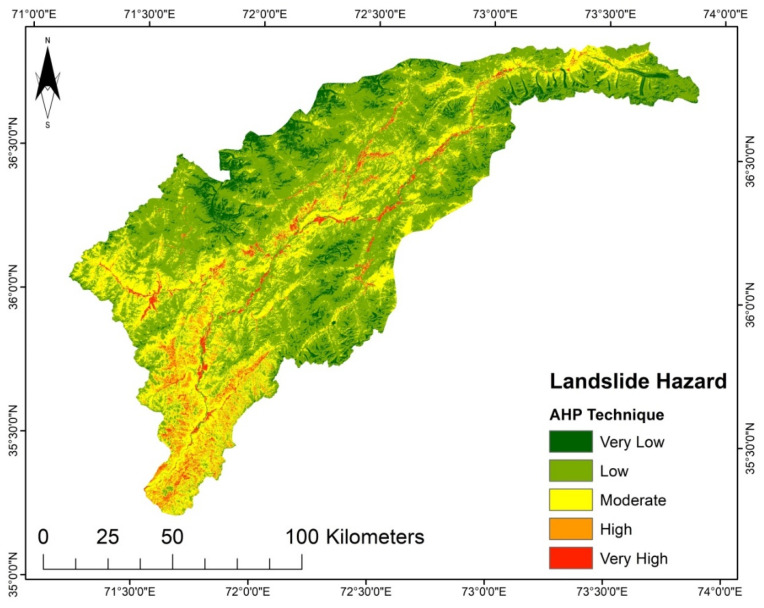
LSM from the AHP technique.

The resulting LSM from the AHP model is exhibited in Figure 7. It shows that the area is generally subject to low susceptibility to landslides except for the central and southwestern regions of the study area, which exhibit high to very high susceptibility, while the LSM based on the TOPSIS model presented in Figure 7 illustrates that the region is dominantly prone to low and moderate landslide susceptibility. The low susceptibility regions are in the north, east, and south of the area. The southwest region shows slightly higher susceptibility. Very high susceptibility can be observed throughout the central part of the area.

### 4.6. Model Validation

There is no technical implication of the landslide susceptibility analysis without validation, so it is essential to evaluate its validity. For the AHP technique, the CR was used for validation purposes. The CR calculated for the AHP technique yielded a value of less than 0.1, which is the requirement for the appropriateness of the technique. Thus, the resulting weights were used for preparing LSM. The comparative closeness coefficient to the ideal solution for each substitute was calculated to validate the TOPSIS technique. The values of the closeness coefficient range from 0 to 1. The best-considered substitute has a score of 1, and the same thing was noticed in this study. Consequently, the obtained weights were used for preparing LSM.

A correlation value was computed for the ML models, SVM, LGR, and LR, between the forecasted and known results using the testing datasets. A higher correlation value indicates greater precision of the established model. The confusion matrix and the validation extent for the ML techniques are represented in Table 5 and Table 6. The accuracy of the LGR model is 82%, and the confusion matrix of the LGR model tells that out of 1012 points, 431 non-landslide and 445 landslide points were predicted accurately. In contrast, the model predicted 75 non-landslide and 61 landslide points incorrectly. The LR model shows an accuracy of 76%. Out of 1012 points, 385 non-landslide and 421 landslide points were accurately predicted. Whereas the model’s incorrectly predicted numbers of non-landslide and landslide points are 121 and 75, respectively, as portrayed in the confusion matrix in Table 5. The accuracy of the SVM model is 85%, which makes it the highest performing model. The SVM model’s confusion matrix shows that out of 1012 points, the number of non-landslide and landslide points that are predicted accurately is 395 and 440, respectively. However, the model’s number of wrongly predicted non-landslide and landslide points is 66 and 111.

## 5. Discussion

While most landslide susceptibility assessment methods are based on GIS technology, many different methods are available to perform the analysis. For the preparation of LSMs, the method used should be highly precise and unpretentious. This study applied delicate ML practices, namely SVM, LR, and LGR, combined with MCDM techniques TOPSIS and AHP to produce LSMs for the Chitral district. The primary step was to compile an inventory of landslides. The pre-landslide and post-landslide results were processed through a CVA to detect the landslides in the same areas. Figure 1c shows the final landslide detection map produced after applying the detection results to detect the landslides. The user input in the CVA detection technique is the three PC images. A final landslide inventory map was produced after integrating the generated information and evaluated by the Kappa coefficient of the agreement. The results were also validated by the site examination of the study region. From zones identified as verified no-landslide out of a total of 193 polygons, 11 polygons (4356 pixels) agreed, and the remaining 181 polygons (11,352 pixels) corresponded to verified landslides.

Sixteen landslide-controlling factors were finalized based on the characteristics of the study region and a review of previously published research to produce the LSMs. Different factors contribute in a unique way to the initiation of a landslide event. The geological and topographical factors initiate a landslide. Yang and Qiao [61] stated that the geological environment is greatly influenced by anthropogenic action. Nevertheless, an open-ended discussion is the accepted strategy for an assortment of landslide conditioning elements [30]. The SRTM DEM with a spatial resolution of 30 m was considered to extract the geomorphological factors, i.e., elevation, gradient, feature, plain curvature, curvature, profile curvature, flow growth, and curvature. LANDSAT-8 satellite images were used to extract the NDVI and NDWI. Published geological maps of Pakistan were used to acquire lithology and fault data. The earthquake activity map was derived from the seismic hazard map of Pakistan. Road networks were derived from Google Earth Images, soil map data was acquired from FAO, and precipitation data was derived from PMD. Multicollinearity analysis was applied to estimate the correlation between the landslide conditioning factors. The outcomes are listed in Table 3, which shows that no collinearity exists between the considered parameters; therefore, all factors were used for landslide prediction models. However, the profile curvature achieved the minimum value of TOL and the maximum value of VIF. The value of VIF for profile curvature is 9.23, which shows that the variation for this factor is high and is considerably correlated. There is a potential multicollinearity for profile curvature because its VIF value is very near to the threshold value. Therefore, its contribution for the ML models is low, and it can potentially cause an error in the developed models. Nevertheless, as the VIF and TOL values for profile curvature are within the acceptable range, profile curvature was considered for this analysis.

In the MCDM techniques, the spatial assessment was carried out to rate and prioritize the contributing datasets. For the ML techniques, the training and testing data analysis helped specify the weights of these contributing datasets. Overall, all the models show that precipitation, elevation, flow, slope, and NDWI have significant impacts. There is very little difference between the influences of these conditioning factors for different models. Thus, it can be concluded that the predictive ability of the applied models measures the conditioning factor’s influence on the LSM. However, conditioning factors behave differently towards different models. Additional analyses are essential to comprehensively explore the influences of all landslide conditioning factors. 

Flow, precipitation, and NDWI are related to water content, and they are among the most significant influencing factors found in the region. The Chitral district is prone to occasional heavy precipitation and considerable snowfall in the winter. Maqsoom and Aslam [88] conducted a study in a part of Northern Pakistan and concluded that the surface water flow, groundwater table, and short-duration high-intensity rainfall events trigger landslides. Flow accumulation helps to estimate the extent to which drainage streams can influence the occurrence of landslides. Streams can disturb the stability of a slope through erosion or by saturating the finer slope material fraction, thus causing a rise in the groundwater table [89,90]. The NDWI measures moisture accumulation at a particular location [89]. Higher soil moisture levels may lead to a higher landslide susceptibility than lower moisture levels. Precipitation plays a dominant role in the occurrence of landslides. Many researchers have put forward numerous investigations regarding rainfall-induced landslides, e.g., [2,61,62].

For the slope stability assessment, two important topographical factors considered in the previous studies in northern Pakistan are elevation and slope [87]. Slope refers to the gradient of ground computed with the horizontal axis and directly impacts the landslides under the action of gravity [88]. The investigated area exhibits an elevation of up to 7700 m above ground and hosts one of the world’s mightiest mountains, Tirich Mir in the Hindu Kush range. Higher elevation areas and steeper slopes have higher landslide susceptibility. Khan and Haneef [48], in an investigation carried out in the Chitral district, mentioned that the population in this high-elevation mountainous area is restricted to tributary–junction fans. Closeness to steep valley slopes leaves these fans susceptible to hydrogeomorphic hazards involving floods, debris flows, and landslides.

The trial-and-error process was used to determine the ideal value of each operator for the ML models to obtain the maximum estimation performance. The landslide inventory was split between testing and training datasets to construct the models. The ML techniques were implemented using the R programming language. Both landslide and non-landslide locations were used to implement the ML techniques to produce the LSMs [36,91]. The models are applied to calculate the correlation value, which evaluates the functioning of the models [91]. The LGR, LR, and SVM models were constructed and justified using relevant factors through training and validation datasets. Additionally, the models were formed using a 10-fold cross-validation process to decrease or remove the change and overfitting. 

The LSMs for the research area were prepared by exploiting the weights of the datasets obtained from TOPSIS, AHP, LR, SVM, and LGR models. Finally, based on the similar interval technique, all the LSMs of the study area were classed into five susceptibility categories (very high, high, moderate, low, and very low) based on the Natural Break classification system. The final low susceptibility index value was classified as very low, and the high susceptibility index value was classified as very high. The in-between values were classified accordingly. Natural Break classification is a general function of ArcGIS–like most GIS systems—and can be applied for the landslide susceptibility classification of any region, although the absolute susceptibility index values will vary from region to region.

According to the LSM (Figure 7) derived from SVM, the areas with elevated steep slopes and high precipitation are highly susceptible to landslides, while the upper north region and lower part fall into the very low to moderate susceptibility zone because of their gentle slope. Contrary to SVM, the LR model was observed to be less complex for training data selection. Based on the landslide inventory data, the LR model strived to fit a linear location and might place the landslide sites into the high and very high susceptibility classes. Likewise, the spatial distribution of hazard zones for the LR model susceptibility map is similar to those based on the other models, but they vary in proportions. The LSM derived from the LR shows that the areas with high flow accumulation and high moisture content in the soil are highly susceptible to landslides. Still, a significant part of the upper north region falls into very low to low susceptibility zones. 

**Figure 8:**
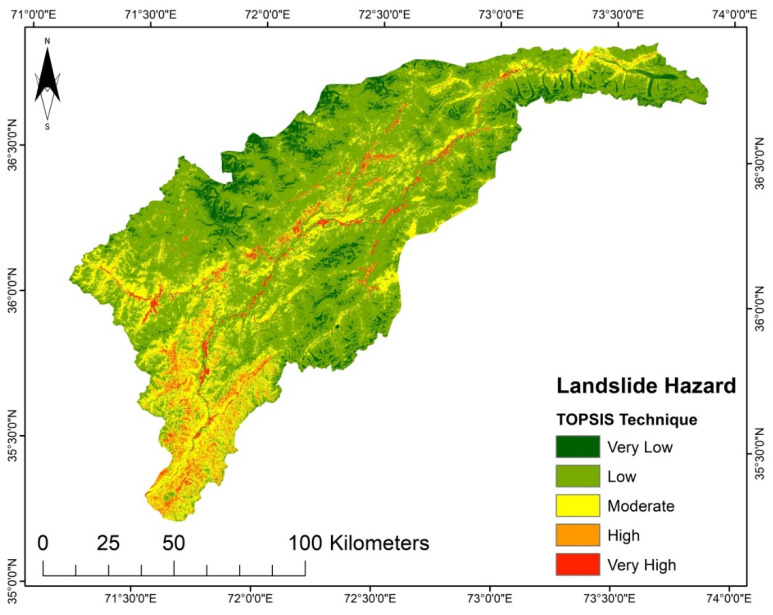
LSM from the TOPSIS technique.

It was determined that the LGR model undoubtedly highlights the interrelation between landslides and instability factors. Besides the SVM and LR, the LGR approach was used due to data availability, and because it is an approach that does not take much time. The LSM derived from the LGR method indicates that the areas with steep slopes and high precipitation are highly susceptible. The upper north region has a very low susceptibility (Figure 4). Though the AHP technique is primarily based on professional judgment, it is supposed that landslide conditioning factors are not impartial [17]. The LSM obtained by AHP shows the high susceptibility regions in areas with steep slopes, high precipitation, and high moisture content, such as in the study area’s middle and lower regions (Figure 8). According to the LSM derived from TOPSIS, a significant portion of the area is classed as low to very low susceptibility. Only the lower southwest and a few central regions show a high to very high susceptibility (Figure 8).

For each of the produced LSM resulting from the practiced models, the categorized five susceptibility groups vary in their percentages and positions in the considered region. The areas in km^2^ and the proportions of all the zones of the susceptibility classes are given in Table 7. The SVM and AHP models have higher area percentages in a very high susceptibility class than the other models. For SVM it is 7.53% (1091.17 km^2^), and for AHP it is 7.31% (1059.29 km^2^) of the study region that falls under the very high susceptibility class. Furthermore, AHP and TOPSIS have the most incredible area in the low susceptibility class, representing a 9.80% (1420.12 km^2^) and 9.18% (1330.27 km^2^) portion of the investigation region, respectively. However, with an area of 45.18% (6547.03 km^2^) and 43.37% (6284.75 km^2^), the LGR and LR models are the dominant ones in the intermediate susceptibility class. Thus, it can be concluded that each technique reacts differently to the conditioning factors and the research area characteristics.

ML models can examine enormous volumes of data and uncover specific trends and patterns that would not be apparent to experts driving MCDM techniques. Moreover, ML models are great at handling multi-variety and multi-dimensional data. They can do this under uncertain or dynamic environments. However, one drawback of ML models is that they require enormous datasets to train on. These should be unbiased, complete, and of good quality. Nevertheless, trading this drawback of massive data requirements for better prediction accuracy is wise when dealing with disastrous modeling hazards. 

The known landslide areas are utilized to validate the landslide susceptibility map. The established data on landslide positions were correlated with the landslide hazard maps to carry out the accuracy assessment. Overall, vital training data selection results were revealed by the spatial distribution of the landslide susceptibility zones. The results confirmed acceptable conformity between the hazard maps and the previously presented data on landslide positions, as can be seen from Table 8. For the landslide susceptibility analysis, the SVM model achieved the maximum implementation accuracy with 88%, followed by the LR model (84%), AHP model (81%), TOPSIS model (79%), and lastly, the LGR model (78%). Even though the used models in this research yielded satisfactory results, it must be noted that the landslide inventory map directly affects the reliability of the products.

## 6. Conclusions

This study employed two MCDM methods (AHP and TOPSIS) and three ML models (LGR, LR, and SVM) to map landslide susceptibility in northern Pakistan’s Chitral district. A landslide inventory analysis was performed and validated. Moreover, different methods for susceptibility mapping were developed and validated in this investigation. Based on earlier studies and the study area’s landslide inventory (training and validation data), sixteen landslide conditioning factors were used. Out of the total operated samples (1012), 506 were non-landslide, and 506 landslide locations were used to calculate the factor weights based on SVM, LGR, and LR. For AHP and TOPSIS, their specified procedures were used to calculate the weights. The outcomes show that elevation, flow accumulation, NDWI, precipitation, and slope are the main conditioning factors. All the conditioning factors were used according to their derived weights to prepare LSMs through a weighted overlay technique in ArcGIS. The results showed that the performance of all models was relatively decent. However, the SVM yielded slightly higher accuracy than the other models. The LSMs are a vital way to outline landslide-prone areas. Therefore, the outcomes of this research can be considered the primary source of information used by engineers, planners, decision-makers, and researchers for proper landslide management in the considered region and other regions with similar geo-environmental features.

## Figures and Tables

**Table 1:** Threshold values and change ratio resulting from the threshold definition.

Change Detection Method	Image Input	Threshold Method	Threshold Value	Change Ratio (%)
CVA	PC	Statistic	>59.326	1.538
CVA	PC	Secant	>29.761	8.237

**Table 2:** Change ratio resulting from the landslide detection and accuracy assessment.

Change Detection Method	Image Input	Threshold Method	Change Ratio (%)	Mean Omission Error	Mean Commission Error	Kappa Coefficient of Agreement
CVA	PC	Statistic	0.43	22.45	15.34	63.453
CVA	PC	Secant	2.73	13.43	11.03	78.483

**Table 3:** Outcomes of the multicollinearity analysis.

Factor	TOL	VIF
Aspect	0.174	5.74
Curvature	0.611	1.64
Earthquake activity	0.567	1.76
Elevation	0.460	2.17
Flow accumulation	0.511	1.96
Lithology	0.463	2.16
NDVI	0.380	2.63
NDWI	0.480	2.09
Plane Curvature	0.134	7.44
Precipitation	0.585	1.71
Profile Curvature	0.108	9.23
Slope	0.216	4.64
Faults	0.244	4.09
Roads	0.407	2.46
Soil	0.273	3.66
Land use	0.309	3.24

**Table 4:** Resulting weights of used conditioning factors from different techniques.

Dataset	SVM	LGR	LR	AHP	TOPSIS
Aspect	4	3	5	7	3
Curvature	7	9	8	4	7
Earthquake activity	3	4	3	5	6
Elevation	9	8	9	6	8
Flow accumulation	9	8	10	7	9
Lithology	7	6	6	8	6
NDVI	4	7	5	6	8
NDWI	7	9	10	9	6
Plane Curvature	3	7	6	5	4
Precipitation	11	9	7	10	13
Profile Curvature	3	2	2	1	2
Slope	10	9	8	11	7
Faults	7	3	4	4	3
Roads	3	3	3	3	3
Soil	6	5	7	6	6
Land use	7	8	7	8	9
Total	100	100	100	100	100

**Table 5:** Confusion Matrix for ML techniques.

Confusion matrix for the LGR model		
	0	1
0	431	61
1	75	445
Confusion matrix for the LR model		
	0	1
0	385	85
1	121	421
Confusion matrix for the SVM model		
	0	1
0	395	111
1	66	440

**Table 6:** Validation of ML models.

Model Type	Validation
LGR	82%
LR	76%
SVM	85%

**Table 7:** Division of landslide susceptibility classes.

Models	Area	Susceptibility Class				
		Very Low	Low	Moderate	High	Very High
LGR	km^2^	678.18	3406.8	6284.75	3211.2	910.03
	%	4.68	23.51	43.37	22.16	6.28
LR	km^2^	375.32	2517.1	6547.03	4271.9	779.62
	%	2.59	17.37	45.18	29.48	5.38
SVM	km^2^	246.35	1904.1	6136.94	5112.4	1091.17
	%	1.70	13.14	42.35	35.28	7.53
AHP	km^2^	1420.12	3787.9	5306.6	2917	1059.29
	%	9.80	26.14	36.62	20.13	7.31
TOPSIS	km^2^	1330.27	4553.1	5087.79	2772.1	747.74
	%	9.18	31.42	35.11	19.13	5.16

**Table 8:** Landslide susceptibility map accuracy.

Models	Accuracy
LGR	78%
LR	84%
SVM	88%
AHP	81%
TOPSIS	79%

## Data Availability

The data that support the findings of this study are available on request from the authors.

## Abbreviations

LSMs	Landslide susceptibility maps
LGR	Logistic regression
LR	Linear regression
AHP	Analytical hierarchy process
AHP	Support vector machines
AHP	Technique for order of preference by similarity to ideal solution
ML	Machine learning
ML	Multi-criteria decision-making
ML	Change vector analysis
ML	Normalized difference vegetation index
NDWI	Normalized difference wetness index
PMD	Pakistan meteorological department
STRM	Shuttle Radar Topography Mission
DEM	Digital elevation model
GIS	Geographical information systems

## References

[1] Chen W., Shahabi H., Zhang S., Khosravi K., Shirzadi A., Chapi K., Pham B.T., Zhang T., Zhang L., Chai H. (2018). Landslide Susceptibility Modeling Based on GIS and Novel Bagging-Based Kernel Logistic Regression. Appl. Sci..

[2] Thai P.B., Bui D.T., Dholakia M.B., Prakash I., Pham H.V. (2016). A comparative study of least square support vector machines and multiclass alternating decision trees for spatial prediction of rainfall-induced landslides in a tropical cyclones area. Geotech. Geol. Eng..

[3] Feizizadeh B., Blaschke T. (2014). An uncertainty and sensitivity analysis approach for GIS-based multicriteria landslide susceptibility mapping. Int. J. Geogr. Inf. Sci..

[4] Solmaz A., Balafar M.A., Feizizadeh B., Sangar A. B., Samadzamini K. (2021). Using hybrid artificial intelligence approach based on a neuro-fuzzy system and evolutionary algorithms for modeling landslide susceptibility in East Azerbaijan Province, Iran. Earth Sci. Inform..

[5] Sajid A., Biermanns P., Haider R., Reicherter K. (2019). Landslide susceptibility mapping by using a geographic information system (GIS) along the China–Pakistan Economic Corridor (Karakoram Highway), Pakistan. Nat. Hazards Earth Syst. Sci..

[6] Abolfazl J., Panahi M., Mafi-Gholami D., Rahmati O., Shahabi H., Shirzadi A., Lee S., Bui D.T., Pradhan B. (2022). Swarm intelligence optimization of the group method of data handling using the cuckoo search and whale optimization algorithms to model and predict landslides. Appl. Soft Comput..

[7] Saeedeh E., Amiri M., Sãdhasivam N., Pourghasemi H.R. (2020). Comparison of new individual and hybrid machine learning algorithms for modeling and mapping fire hazard: A supplementary analysis of fire hazard in different counties of Golestan Province in Iran. Nat. Hazards.

[8] Saeedeh E. (2021). Fire of Iranian forests, consequences, opposition methods and solutions. Hum. Environ..

[9] Xiaojing W., Huang F., Fan X., Shahabi H., Shirzadi A., Bian H., Ma X., Lei X., Chen W. (2022). Landslide susceptibility modeling based on remote sensing data and data mining techniques. Environ. Earth Sci..

[10] Ngo P.T., Panahi M., Khosravi K., Ghorbanzadeh O., Kariminejad N., Cerda A., Lee S. (2021). Evaluation of deep learning algorithms for national scale landslide susceptibility mapping of Iran. Geosci. Front..

[11] Conforti M., Ietto F. (2021). Modeling shallow landslide susceptibility and assessment of the relative importance of predisposing factors, through a GIS-based statistical analysis. Geosciences.

[12] Reichenbach P., Rossi M., Malamud B.D., Mihir M., Guzzetti F. (2018). A review of statisticallybased landslide susceptibility models. Earth Sci. Rev..

[13] Jacek M. (2004). GIS-based land-use suitability analysis: A critical overview. Prog. Plan..

[14] Omarzadeh D., Pourmoradian S., Feizizadeh B., Khallaghi H., Sharifi A., Kamran K.V. (2022). A GIS-Based Multiple Ecotourism Sustainability Assessment of West Azerbaijan Province Iran. J. Environ. Plan. Manag..

[15] Ghorbanzadeh O., Pourmoradian S., Blaschke T., Feizizadeh B. (2019). Mapping Potential Nature-Based Tourism Areas by Applying GIS-Decision Making Systems in East Azerbaijan Province. Iran. J. Ecotourism.

[16] Neaupane K.M., Piantanakulchai M. (2006). Analytic network process model for landslide hazard zonation. Eng. Geol..

[17] Rao R.V., Davim J.P. (2008). A decision-making framework model for material selection using a combined multiple attribute decision-making method. Int. J. Adv. Manuf. Technol..

[18] Zare M., Pourghasemi H.R., Vafakhah M., Pradhan B. (2013). Landslide susceptibility mapping at Vaz Watershed (Iran) using an artificial neural network model: A comparison between multilayer perceptron (MLP) and radial basic function (RBF) algorithms. Arab. J. Geosci..

[19] Zhang W., Li H., Han L., Chen L., Wang L. (2022). Slope stability prediction using ensemble learning techniques: A case study in Yunyang County, Chongqing, China. J. Rock Mech. Geotech. Eng..

[20] Zhang W., Zhang R., Wu C., Goh A.T., Wang L. (2022). Assessment of basal heave stability for braced excavations in anisotropic clay using extreme gradient boosting and random forest regression. Undergr. Space.

[21] Zhang W., Zhang R., Wu C., Goh A.T., Lacasse S., Liu Z., Liu H. (2020). State-of-the-art review of soft computing applications in underground excavations. Geosci. Front..

[22] Chen W., Peng J., Hong H., Shahabi H., Pradhan B., Liu J., Zhu A.X., Pei X., Duan Z. (2018). Landslide susceptibility modelling using GIS-based machine learning techniques for Chongren County, Jiangxi Province, China. Sci. Total Environ..

[23] Pourghasemi H.R., Gayen A., Park S., Lee C.W., Lee S. (2018). Assessment of landslide-prone areas and their zonation using logistic regression, logitboost, and naïvebayes machine-learning algorithms. Sustainability.

[24] Raja N.B., Çiçek I., Türkoğlu N., Aydin O., Kawasaki A. (2017). Landslide susceptibility mapping of the Sera River Basin using logistic regression model. Nat. Hazards.

[25] Othman A.A., Gloaguen R., Andreani L., Rahnama M. (2018). Improving landslide susceptibility mapping using morphometric features in the Mawat area, Kurdistan Region, NE Iraq: Comparison of different statistical models. Geomorphology.

[26] Chen T., Niu R., Jia X. (2016). A comparison of information value and logistic regression models in landslide susceptibility mapping by using GIS. Environ. Earth Sci..

[27] Akgün A., Türk N. (2011). Mapping erosion susceptibility by a multivariate statistical method: A case study from the Ayvalık region, NW Turkey. Comput. Geosci..

[28] Guzzetti F., Reichenbach P., Ardizzone F., Cardinali M., Galli M. (2006). Estimating the quality of landslide susceptibility models. Geomorphology.

[29] Rahmati O., Pourghasemi H.R. (2017). Identification of critical flood prone areas in data-scarce and ungauged regions: A comparison of three data mining models. Water Resour. Manag..

[30] Tien Bui D., Tuan T.A., Klempe H., Pradhan B., Revhaug I. (2016). Spatial prediction models for shallow landslide hazards: A comparative assessment of the efficacy of support vector machines, artificial neural networks, kernel logistic regression, and logistic model tree. Landslides.

[31] Bai S., Lü G., Wang J., Zhou P., Ding L. (2011). GIS-based rare events logistic regression for landslide-susceptibility mapping of Lianyungang, China. Environ. Earth Sci..

[32] Domínguez-Cuesta M.J., Jiménez-Sánchez M., Berrezueta E. (2007). Landslides in the Central Coalfield (Cantabrian Mountains, NW Spain): Geomorphological features, conditioning factors and methodological implications in susceptibility assessment. Geomorphology.

[33] Pradhan B., Youssef A., Varathrajoo R. (2010). Approaches for delineating landslide hazard areas using different training sites in an advanced artificial neural network model. Geo-Spat. Inf. Sci..

[34] Peng L., Niu R., Huang B., Wu X., Zhao Y., Ye R. (2014). Landslide susceptibility mapping based on rough set theory and support vector machines: A case of the Three Gorges area, China. Geomorphology.

[35] Aghdam I.N., Varzandeh M.N.H., Pradhan B. (2016). Landslide susceptibility mapping using an ensemble statistical index (Wi) and adaptive neuro-fuzzy inference system (ANFIS) model at Alborz Mountains (Iran). Environ. Earth Sci..

[36] Chen W., Pourghasemi H.R., Kornejady A., Zhang N. (2017). Landslide spatial modeling: Introducing new ensembles of ANN, MaxEnt, and SVM machine learning techniques. Geoderma.

[37] Dehnavi A., Aghdam I.N., Pradhan B., Varzandeh M.H. (2015). A new hybrid model using step-wise weight assessment ratio analysis (SWARA) technique and adaptive neuro-fuzzy inference system (ANFIS) for regional landslide hazard assessment in Iran. Catena.

[38] Kanungo D.P., Arora M.K., Sarkar S., Gupta R.P. (2006). A comparative study of conventional, ANN black box, fuzzy and combined neural and fuzzy weighting procedures for landslide susceptibility zonation in Darjeeling Himalayas. Eng. Geol..

[39] Ghorbanzadeh O., Blaschke T., Aryal J., Gholaminia K. (2020). A new GIS-based technique using an adaptive neuro-fuzzy inference system for land subsidence susceptibility mapping. J. Spat. Sci..

[40] Aslam B., Zafar A., Khalil U. (2021). Correction to: Development of integrated deep learning and machine learning algorithm for the assessment of landslide hazard potential. Soft Comput..

[41] Khosravi K., Panahi M., Golkarian A., Keesstra S.D., Saco P.M., Bui D.T., Lee S. (2020). Convolutional neural network approach for spatial prediction of flood hazard at national scale of Iran. J. Hydrol..

[42] Omarzadeh D., Karimzadeh S., Matsuoka M., Feizizadeh B. (2021). Earthquake Aftermath from Very High-Resolution WorldView-2 Image and Semi-Automated Object-Based Image Analysis (Case Study: Kermanshah, Sarpol-e Zahab, Iran). Remote Sens..

[43] Ghorbanzadeh O., Blaschke T., Gholamnia K., Meena S.R., Tiede D., Aryal J. (2019). Evaluation of different machine learning methods and deep-learning convolutional neural networks for landslide detection. Remote Sens..

[44] Kolb H. (1994). Abflußverhalten von Flüssen in den Hochgebirgen Nordpakistans. Physisch-geographische Beiträge zu den Hochgebirgsräumen Nordpakistans und der Alpen. Beitr. U. Mat. Z. Reg. Geogr.

[45] Huserodt K. (2008). Change of climate in the Hindu Kush region-facts, trends, and necessary observations of the environment. Proceedings of the Third International Hindu Kush Cultural Conference.

[46] Kamp U., Haserodt K., Shroder J.F. (2004). Quaternary landscape evolution in the eastern Hindu Kush, Pakistan. Geomorphology.

[47] Roohi R., Ashraf R., Naz R., Hussain S.A., Chaudhry M.H. (2005). Inventory of Glaciers and Glacial Lakes Outburst Floods (GLOFs) Affected by Global Warming in the Mountains of Himalayan Region, Indus Basin, Pakistan Himalaya.

[48] Asif Khan M., Haneef M., Khan A.S., Tahirkheli T. (2013). Debris-flow hazards on tributary junction fans, Chitral, Hindu Kush Range, northern Pakistan. J. Asian Earth Sci..

[49] Hafeez S., Waqas A., Azam S., Khan S. (2019). Evaluation of landslide hazards at Herth, Chitral, Pakistan. Innov. Infrastruct. Solut..

[50] Aslam B., Khalil U., Saleem M., Maqsoom A., Khan E. (2021). Effect of multiple climate change scenarios and predicted land-cover on soil erosion: A way forward for the better land management. Environ. Monit. Assess..

[51] Hotelling H. (1933). Analysis of a complex of statistical variables into principal components. J. Educ. Psychol..

[52] Ramos-Bernal R.N., Vázquez-Jiménez R., Romero-Calcerrada R., Arrogante-Funes P., Novillo C.J. (2018). Evaluation of unsupervised change detection methods applied to landslide inventory mapping using ASTER imagery. Remote Sens..

[53] Ferrero S., Palacio M., Campanella O.R. (2002). Análisis de componentes principales en teledetección. Consideraciones estadísticas para optimizar su interpretación. Rev. Teledetección.

[54] Lorena R.B., Santos J.D., Shimabukuro Y.E., Brown I.F., Kux H.J. (2002). A change vector analysis technique to monitor land use/land cover in sw Brazilian amazon: Acre state. PECORA 15-Integr. Remote Sens. Glob. Reg. Local Scale.

[55] Roemer H., Kaiser G., Sterr H., Ludwig R. (2010). Using remote sensing to assess tsunami-induced impacts on coastal forest ecosystems at the Andaman Sea coast of Thailand. Nat. Hazards Earth Syst. Sci..

[56] Lüdeke M.K., Ramage P.H., Kohlmaier G. (1996). The use of satellite NDVI data for the validation of global vegetation phenology models: Application to the Frankfurt Biosphere Model. Ecol. Model..

[57] Malila W.A. (1980). Change vector analysis: An approach for detecting forest changes with Landsat. LARS Symposia.

[58] Chen J., Gong P., He C., Pu R., Shi P. (2003). Land-use/land-cover change detection using improved change-vector analysis. Photogramm. Eng. Remote Sens..

[59] Cohen J. (1960). A coefficient of agreement for nominal scales. Educ. Psychol. Meas..

[60] Vázquez-Jiménez R., Ramos-Bernal R.N., Romero-Calcerrada R., Arrogante-Funes P., Tizapa S.S., Novillo C.J., Travieso-Gonzalez C. (2018). Thresholding algorithm optimization for change detection to satellite imagery. Color. Image Process.

[61] Nasir S.M., Kamran K.V., Blaschke T., Karimzadeh S. (2022). Change of Land Use/Land Cover in Kurdistan Region of Iraq: A Semi-Automated Object-Based Approach. Remote Sens. Appl. Soc. Environ..

[62] Yang Z., Qiao J., Uchimura T., Wang L., Lei X., Huang D. (2017). Unsaturated hydro-mechanical behaviour of rainfall-induced mass remobilization in post-earthquake landslides. Eng. Geol..

[63] Tsangaratos P., Ilia I., Hong H., Chen W., Xu C. (2017). Applying Information Theory and GIS-based quantitative methods to produce landslide susceptibility maps in Nancheng County, China. Landslides.

[64] Youssef A.M., Pourghasemi H.R., El-Haddad B.A., Dhahry B.K. (2016). Landslide susceptibility maps using different probabilistic and bivariate statistical models and comparison of their performance at Wadi Itwad Basin, Asir Region, Saudi Arabia. Bull. Eng. Geol. Environ..

[65] Hong H.S.A., Naghibi H.R., Pradhan P.B. (2016). GIS-based landslide spatial modeling in Ganzhou City, China. Arab J. Geosci..

[66] Chen W., Zhang S., Li R., Shahabi H. (2018). Performance evaluation of the GIS-based data mining techniques of best-first decision tree, random forest, and naïve Bayes tree for landslide susceptibility modeling. Sci. Total Environ..

[67] O’brien R.M. (2007). A caution regarding rules of thumb for variance inflation factors. Qual. Quant..

[68] Colkesen I., Sahin E.K., Kavzoglu T. (2016). Susceptibility mapping of shallow landslides using kernel-based Gaussian process, support vector machines and logistic regression. J. Afr. Earth Sci..

[69] Wang L., Wu C., Tang L., Zhang W., Lacasse S., Liu H., Gao L. (2020). Efficient reliability analysis of earth dam slope stability using extreme gradient boosting method. Acta Geotech..

[70] Zhang W., Wu C., Zhong H., Li Y., Wang L. (2021). Prediction of undrained shear strength using extreme gradient boosting and random forest based on Bayesian optimization. Geosci. Front..

[71] Irigaray C., Fernández T., El Hamdouni R., Chacón J. (2007). Evaluation and validation of landslide-susceptibility maps obtained by a GIS matrix method: Examples from the Betic Cordillera (southern Spain). Nat. Hazards.

[72] Lee S., Sambath T. (2006). Landslide susceptibility mapping in the Damrei Romel area, Cambodia using frequency ratio and logistic regression models. Environ. Geol..

[73] Yesilnacar E., Topal T. (2005). Landslide susceptibility mapping: A comparison of logistic regression and neural networks methods in a medium scale study, Hendek region (Turkey). Eng. Geol..

[74] Vapnik V. (1998). The support vector method of function estimation. Nonlinear Modeling.

[75] Cortes C., Vapnik V. (1995). Support-vector networks. Mach. Learn..

[76] Brenning A. (2005). Spatial prediction models for landslide hazards: Review, comparison and evaluation. Nat. Hazards Earth Syst. Sci..

[77] Kavzoglu T., Sahin E.K., Colkesen I. (2014). Landslide susceptibility mapping using GIS-based multi-criteria decision analysis, support vector machines, and logistic regression. Landslides.

[78] Mountrakis G., Im J., Ogole C. (2011). Support vector machines in remote sensing: A review. ISPRS J. Photogramm. Remote Sens..

[79] Saaty T. (1980). The Analytic Hierarchy Process.

[80] Saaty T.L., Vargas L.G. (2001). How to make a decision. Models, Methods, Concepts & Applications of the Analytic Hierarchy Process.

[81] Ching L.H., Yoon P. (1981). Multiple Attribute Decision Making. Lecture Notes in Economics and Mathematical Systems.

[82] Ameri A.A., Pourghasemi H.R., Cerda A. (2018). Erodibility prioritization of sub-watersheds using morphometric parameters analysis and its mapping: A comparison among TOPSIS, VIKOR, SAW, and CF multi-criteria decision making models. Sci. Total Environ..

[83] Opricovic S., Tzeng G.-H. (2004). Compromise solution by MCDM methods: A comparative analysis of VIKOR and TOPSIS. Eur. J. Oper. Res..

[84] Basharat M., Shah H.R., Hameed N. (2016). Landslide susceptibility mapping using GIS and weighted overlay method: A case study from NW Himalayas, Pakistan. Arab. J. Geosci..

[85] Shit P.K., Bhunia G.S., Maiti R. (2016). Potential landslide susceptibility mapping using weighted overlay model (WOM). Modeling Earth Syst. Environ..

[86] Bachri S., Shresta R.P. (2010). Landslide hazard assessment using analytic hierarchy processing (AHP) and geographic information system in Kaligesing mountain area of Central Java Province Indonesia. Annu. Int. Work. Expo Sumatra Tsunami.

[87] Intarawichian N., Dasananda S. (2010). Analytical Hierarchy Process for landslide suscpetibility mapping in lower Mae Chaem watershed, northern Thiland. Suranaree J. Sci. Technol..

[88] Maqsoom A., Aslam B., Khalil U., Kazmi Z.A., Azam S., Mehmood T., Nawaz A. (2021). Landslide susceptibility mapping along the China Pakistan Economic Corridor (CPEC) route using multi-criteria decision-making method. Modeling Earth Syst. Environ..

[89] Rahim I., Ali S.M., Aslam M. (2018). GIS Based landslide susceptibility mapping with application of analytical hierarchy process in District Ghizer, Gilgit Baltistan Pakistan. J. Geosci. Environ. Prot..

[90] Ballabio C., Sterlacchini S. (2012). Support vector machines for landslide susceptibility mapping: The Staffora River Basin case study, Italy. Math. Geosci..

[91] Chen W., Pourghasemi H.R., Naghibi S.A. (2018). Prioritization of landslide conditioning factors and its spatial modeling in Shangnan County, China using GIS-based data mining algorithms. Bull. Eng. Geol. Environ..

